# The value of adrenal venous sampling in unveiling the conundrum of double unilateral adrenal adenomas in a case of primary hyperaldosteronism

**DOI:** 10.1177/2050313X261487243

**Published:** 2026-09-01

**Authors:** Maab F. Elhaj, Elabbass A. Abdelmahmuod, Stephen F. Beer, Tarik Elhadd

**Affiliations:** 1 Endocrine Section, Department of Medicine, 36977 Hamad Medical Corporation, Doha, Qatar; 2 Department of Internal Medicine, 36977 Hamad Medical Corporation, Doha, Qatar

**Keywords:** adrenal, adenoma, hyperaldosteronism, venous sampling, hypertension, adrenal hyperplasia

## Abstract

Primary hyperaldosteronism (PHA) is a cause of secondary hypertension, often due to adrenal adenoma. The diagnosis and management of multiple unilateral adrenal adenomas in PHA represent a unique challenge. Adrenal venous sampling (AVS) has become a pivotal diagnostic tool in these cases, providing critical information for accurate localization of aldosterone production. Here, we describe a rare case of a 52-year-old female with a history of resistant essential hypertension who presented with multiple unilateral adrenal adenomas concomitant with adrenal hyperplasia in a case of primary hyperaldosteronism. The use of AVS in localization of aldosterone production was the determining factor in deciding the ultimate management of the patient, leading to successful follow-up on medical therapy with spironolactone.

## Introduction

Angiotensin II and plasma potassium concentration are the two main regulators of the aldosterone secretion; however, in primary hyperaldosteronism, these factors are not suppressed by sodium loading, and aldosterone production is abnormally high for the sodium status. An excessive amount of aldosterone is produced, which can lead to hypertension, cardiovascular disease, sodium retention, decreased plasma renin, and increased potassium excretion, which can result in hypokalaemia.^1^ Common causes of primary hyperaldosteronism (PHA) include adrenal adenoma, unilateral or bilateral adrenal hyperplasia, adrenal carcinoma, and hereditary disorders such as familial hyperaldosteronism. Jerome W Conn was the first to report on primary hyperaldosteronism, the first to characterise PHA, its incidence, and its course of therapy, and the condition is often referred to as Conn’s syndrome.^2^

Adrenal venous sampling (AVS) is the gold standard for differentiating unilateral from bilateral primary hyperaldosteronism. This procedure involves catheterizing both adrenal veins and the inferior vena cava to measure aldosterone and cortisol levels. The selectivity index, calculated as the ratio of cortisol in the adrenal vein to that in the inferior vena cava, confirms successful catheterization.^3^ A lateralization index, the ratio of the aldosterone/cortisol ratio from one adrenal vein to the other, is then used to determine the source of aldosterone excess. While highly accurate, AVS is an invasive and technically demanding procedure, requiring experienced interventional radiologists.^4^

The management of PHA must be guided by the lateralization of the source of the increased aldosterone secretion.^5^ Separating unilateral from bilateral disease is crucial because hypokalemia normalizes after unilateral adrenalectomy in all patients with unilateral adrenal hyperplasia or aldosterone-producing adenoma; and hypertension also improves nearly in all and is cured in 30–60% of these patients.^6,7^ Unusually, unilateral or bilateral adrenalectomy will reduce hypertension in patients with bilateral idiopathic hyperaldosteronism (bilateral idiopathic adrenal hyperplasia) and glucocorticoid-remediable aldosteronism (GRA) and medical therapy is the treatment of choice.^8–10^ If the patient refuses surgery or is not a candidate, medical treatment for unilateral conditions may be pursued. For most patients, imaging does not confidently identify aldosterone-producing adenomas from non-functioning adrenal incidentalomas; hence, AVS is the most accurate way to differentiate between unilateral and bilateral forms of primary Hyperaldosteronism (PHA).^11^ In this case report we aimed to point out the critical use of adrenal venous sampling (AVS) for precise localization of aldosterone production in a patient who had proven primary hyperaldosteronism who’s imaging a showed multiple unilateral adrenal adenoma. We also include a comprehensive literature review of the subject.

### Case presentation

The reporting of this study conforms to CARE guidelines (36). A 52-year-old patient was diagnosed with essential hypertension in 2016. She had a rather resistant course requiring three medications to control her blood pressure. She had some investigations for secondary hypertension by the internist who saw her initially in 2016, but no tests for hyperaldosteronism. Early in 2023 she was with palpitations by the cardiology team, who felt her BP control is not optimal. A thiazide diuretic, indapamide was added to her other antihypertensive agents, which resulted in significant drop in potassium levels. At this point the issue of secondary hypertension was considered. Workup showed no evidence of pheochromocytoma or hypercortisolism but indicated an aldosterone level of 283 pmol/l (normal ranges 70–350 pmol/L), plasma renin activity of 0.26 ng/ml/hr (Normal range 0.2 – 1.6 ng/mL/hr), and aldosterone/renin ratio of 39 (N <20).

**Figure 1.**
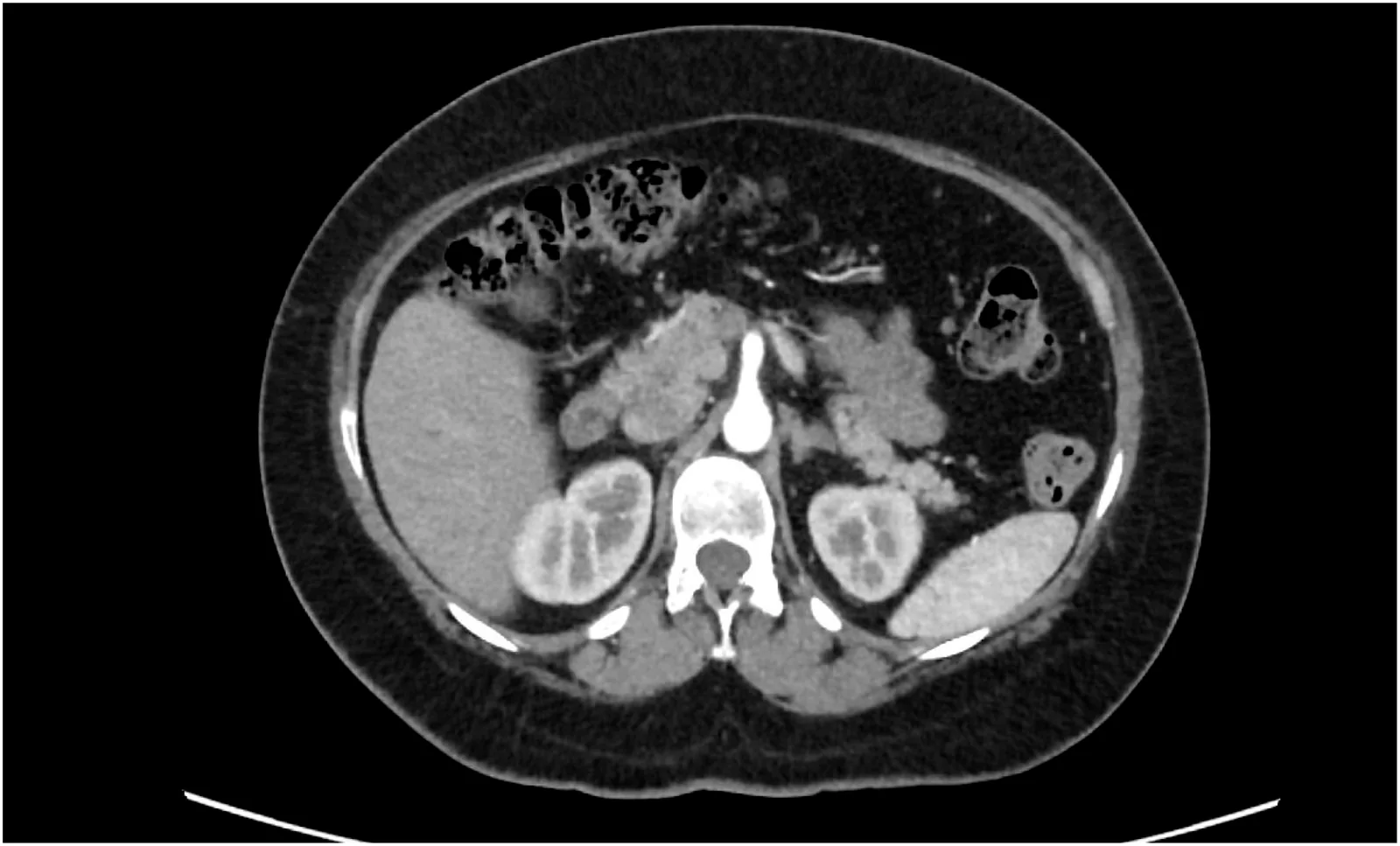
Abdominal CT scan showing two left adrenal lesions (arrows) consistent with adrenal adenomas. Representing CT-abdomen showing two left adrenal lesion seen show features keeping with adrenal adenomas.

A CT scan of the abdomen revealed two left adrenal lesions, measuring 14 and 15 mm in maximum diameters, which were consistent with adrenal adenomas (Figure 1). She was started on spironolactone and other antihypertensives were stopped. That resulted in a significant improvement of her BP control. In October 2020, the patient was referred to the Mayo Clinic (Rochester, Minnesota, USA) for adrenal venous sampling (AVS). Adrenal venous sampling confirmed that the source of high aldosterone was from both adrenals (Table 1), consistent with mild bilateral hyperplasia. A recommendation was made to continue medical therapy with spironolactone 50mg/day.

**Table 1.** Adrenal venous sampling result and interpretation.

Adrenal vein sampling results and interpretation			
Measurement	Inferior vena cava	Right adrenal vein	Left adrenal vein
Cortisol (nmol/L)	28	436	392
Selectivity Index (Adrenal/IVC cortisol) Note: At least 5:1 with cosyntropin infusion and 3:1 without infusion.	N/A	15.57	14
Aldosterone (Pmol/L)	8.5	270	310
Aldosterone-to-cortisol ratio	N/A	0.61	0.79

IVC: Inferior Vena cava, NA: Not Applicable.

### Definitions

Selectivity Index: The ratio of cortisol concentration in the adrenal vein to that in the inferior vena cava (IVC). A ratio of >3:1 without cosyntropin stimulation or >5:1 with cosyntropin stimulation indicates successful cannulation of the adrenal vein^12^.

Lateralization Index: The ratio of the aldosterone-to-cortisol ratio of the dominant side to the non-dominant side. A ratio >4:1 is typically used to indicate unilateral disease.^13^

### Follow-up

Following the AVS results confirming bilateral aldosterone secretion, a recommendation was made to continue medical therapy. The patient was treated with spironolactone 50mg daily, which resulted in a significant improvement in her blood pressure control and normalization of her serum potassium. The key clinical and biochemical parameters at baseline and during follow-up on medical therapy are summarized in Table 2.

**Table 2.** Baseline and follow-up clinical and biochemical parameters.

Parameter	Baseline	Follow-up (on Spironolactone)
Blood Pressure (mmHg)	165/105	130/85
Serum K+ (mmol/L)	3.1	4.5
eGFR/Cr (ml/min/1.73m2)	85	80
Cortisol (nmol/L)	350	340
Renin (ng/ml/hr)	0.26	1.5
Aldosterone (pmol/L)	283	450

## Discussion

With an estimated prevalence of 5% to 10% in unselected populations and up to 20% in patients with resistant hypertension, primary hyperaldosteronism is a common cause of secondary hypertension.^14–17^ The disease is caused by either unilateral (limited to one adrenal) or bilateral excess aldosterone production, and the two types are best treated with a unilateral adrenalectomy or a mineralocorticoid receptor antagonist, respectively.^18,19^ The most prevalent surgically treatable cause of hypertension is unilateral primary hyperaldosteronism, and the percentage of patients who get clinical remission following surgery varies greatly.^20,21^ The coexistence of adrenal hyperplasia and multiple adenomas in our patient suggests a potential common underlying pathophysiology, where a background of hyperplasia may create a permissive environment for the development of discrete adenomas. This interplay warrants further investigation to elucidate the molecular mechanisms driving this phenomenon.

The presence of multiple unilateral adrenal adenomas in primary hyperaldosteronism is a rare but increasingly recognized entity. A review of the literature reveals several case reports and small series that highlight the diagnostic and management challenges associated with this condition (Table 2). These cases underscore the importance of adrenal venous sampling (AVS) in correctly lateralizing the source of aldosterone excess, especially when imaging findings are equivocal or show multiple nodules.^18,22,23^

Double functioning adrenocortical adenomas, occurring in the same gland is an extremely rare condition and difficult to diagnose. Adrenal CT scanning or bilateral adrenal vein sampling (AVS) are employed for the diagnosis.^24^ Adrenal CT is inexpensive and widely accessible, although it is not very accurate in identifying aldosterone-producing adenomas. AVS is costly and calls for a high level of technical proficiency.^25^ As such, its availability is not as broad as that of CT. One benefit of using AVS for CT-identified nodules is the ability to establish a definite nodule. It can also detect aldosterone-producing adenomas below CT’s detection limit. As a result, AVS has become the accepted benchmark for primary aldosteronism subtyping.^26–28^ 38% of instances had a diagnostic discrepancy between CT and AVS, according to a comprehensive analysis that reviewed mostly retrospective research.^29^ Regarding the course of treatment, there is, however, little data to support the superiority of AVS.^30,31^

Two examples of double functional adrenocortical adenomas within the same adrenal gland, resulting in primary aldosteronism were reported by Sapalidis. In one case, the diagnosis was made histologically because magnetic resonance imaging (MRI) was unable to differentiate between the two entities. An adrenalectomy under laparoscopy was performed in each patient. Histopathology reports should be sufficiently detailed to identify unusual lesions such as double adrenocortical adenomas when preoperative imaging investigations are unable to detect them.^24,32^

A 46-year-old woman who suffered two months of paroxysmal hypertension was the subject of a case report by Wei Wang. This patient had bilateral adrenal vein sampling (AVS), as well as clinical, laboratory, and imaging examinations. Particularly, bilateral adrenal adenoma was discovered by magnetic resonance imaging and computed tomography scanning. Furthermore, bilateral AVS was carried out to differentiate between the patient’s unilateral and bilateral adrenal abnormalities. She underwent screening, differential diagnosis, functional testing, many imaging procedures, bilateral adrenal vein sampling (AVS) typing, and ultimately was diagnosed as a case of idiopathic hyperaldosteronism. After treatment with aldosterone antagonists, her condition improved, and her blood pressure was controlled, and potassium levels also returned to normal.^33^

In our report, we highlight the importance of adrenal sampling by describing a patient who had double unilateral adrenal adenomas along with adrenal hyperplasia in the context of biochemically proven primary hyperaldosteronism which helped to guide the management (Table 3).

**Table 3.** Summarizes key features of reported cases of multiple unilateral adrenal adenomas.

Author (Year)	Age/Sex	Number of adenomas	Size of adenomas (mm)	AVS findings	Treatment	Outcome
Sapalidis et al. (2018)	52/F	2	15, 20	Not performed	Laparoscopic adrenalectomy	Resolution of hyperaldosteronism
Wang et al. (2019)	46/F	2 (bilateral)	Not specified	Bilateral aldosterone secretion	Medical therapy	Blood pressure and potassium controlled
Hirono et al. (2005)	58/M	Multiple micronodules	<10	Left-sided aldosterone secretion	Left adrenalectomy	Remission of hyperaldosteronism
Morimoto et al. (1992)	48/F	2	10, 15	Left-sided aldosterone secretion	Left adrenalectomy	Cure of hyperaldosteronism
Our Case	52/F	2	14, 15	Bilateral aldosterone secretion	Medical therapy	Blood pressure and potassium controlled

## Conclusion

This is a very rare case of multiple unilateral adrenal adenomas concomitant with adrenal hyperplasia in a case of primary hyperaldosteronism.^34^ Only handful number of cases have been reported in the literature so far. A subset of patients with bilateral adrenal hyperplasia may have propensity to develop nodules. The true nature of the multiple adrenal adenomas in such context may cause diagnostic difficulty. Here, adrenal venous sampling comes to the fore as the gold standard to resolve such conundrum.

## Data Availability

All data relevant to the case are included in the article, No additional data are available. Patient confidentiality precludes the sharing of raw data beyond what is presented in the report.*
